# Efficacy and safety of DPP-4 inhibitor in the treatment of patients with COVID-19 combined with diabetes mellitus: A protocol for systematic review and meta-analysis

**DOI:** 10.1097/MD.0000000000022592

**Published:** 2020-10-09

**Authors:** Yan Liu, Hongyan Xie, Hong Gao, Chunguang Xie

**Affiliations:** a Hospital of Chengdu University of Traditional Chinese Medicine; b Chengdu University of Traditional Chinese Medicine, Chengdu, Sichuan Province, China.

**Keywords:** COVID-19, diabetes mellitus, DPP-4 inhibitors, meta-analysis, protocol, systematic review

## Abstract

**Background:**

DM is a common chronic metabolic disease. COVID-19 is a large-scale infectious disease. Some studies have shown that DM is an independent risk factor that increases COVID-19 mortality or other adverse outcomes. There is currently no specific and effective drug treatment. More and more people realize that DPP-4 inhibitors may play a huge role in fighting COVID-19 combined with diabetes. However, there is no evidence-based medicine to confirm the effectiveness and safety of DPP-4 inhibitors in the treatment of COVID-19 patients with diabetes. Therefore, we will conduct a systematic review and meta-analysis to synthesize the existing clinical evidence.

**Methods and analysis:**

Electronic databases include CNKI, Wanfang, VIP, CBM database, Cochrane Library, PubMed, Web of Science, EMBASE, etc. We will retrieve each database from December 2019 to September 2020. At the same time, we will look for clinical trial registration and gray literature. This study only included clinical randomized controlled trials. The reviewers independently conduct literature selection, data analysis, quality analysis, and evaluation. The primary outcomes include mortality rate, morbidity, interleukin-6, tumor necrosis factor-alpha, clinical improvement, symptoms improvement, fasting blood glucose, 2-hour postprandial blood glucose, glycosylated hemoglobin, fasting insulin, adverse reactions, etc. Finally, we will conducted a meta-analysis through Review Manager Software version 5.3.

**Results:**

The results will be published in peer-reviewed journals and presented at a relevant conference.

**Conclusion:**

This study will explore the effectiveness and safety of DPP-4 inhibitors in the treatment of COVID-19 patients with diabetes. It will provide evidence-based medical evidence for DPP-4 inhibitors in the treatment of diabetes with COVID-19.

**Registration number:**

INPLASY202090015.

## 1 Introduction

In December 2019, coronavirus disease 2019 (COVID-19) broke out in China and spread rapidly all over the world.^[1]^ As of the time of writing this article, a total of 90,442 cases of COVID-19 have been diagnosed nationwide, 4734 cases have died, and 85,169 cases have been cured. A total of 212 countries and regions overseas have reported 26,215,004 confirmed cases, 867,865 deaths, and 18,440,138 cured. The prevalence of DM is increasing year by year and is closely related to infection. Many studies have found that DM will increase the morbidity and mortality of COVID-19.^[2–5]^ Therefore, patients with diabetes and COVID-19 may require special attention and clinical care.

Dipeptidyl peptidase-4 (DPP-4), also known as CD26, is a multi-expressed glycoprotein.^[6]^ Many studies have shown that membrane-associated human DPP-4, as a functional receptor of Middle East respiratory syndrome coronavirus (MERS-CoV), interacts with MERS-CoV through the spike glycoprotein S1b domain to promote virus entry.^[7]^ Since SARS-CoV-2 and MERS-CoV belong to the same subgenus, and the similar outer membrane spike glycoproteins among the coronavirus,^[8]^ it is speculated that membrane-related human DPP-4 may also be a functional SARS-CoV-2 receptor. Therefore, the research of DPP-4 inhibitors can be used as a new strategic direction to prevent COVID-19. Using existing DPP4 inhibitors (such as sitagliptin, linenegliptin, vildagliptin, etc.) to inhibit the activity of DPP4/CD26 may be an effective weapon to block the host CD26 receptor, thereby blocking SARS CoV- 2 Enter T cells to prevent infection of COVID-19.^[9]^ Furthermore, as a class of oral hypoglycemic agents, DPP-4 inhibitors can effectively reduce glycosylated hemoglobin. Therefore, the researches of DPP-4 inhibitors have exciting potential for diabetic patients infected with COVID-19.

Therefore, this article aims to explore the effectiveness and safety of DPP-4 inhibitors in the treatment of COVID-19 patients with DM. This result may provide a new basis for the clinical treatment of COVID-19 combined with DM.

## 2 Methods and analysis

### 2.1 Study registration

We have completed the registration of the systematic review protocol on the INPLASY website as INPLASY202090015 (https://inplasy.com/inplasy-2020-9-0015/). It is reported on the basis of Cochrane Handbook for Systematic Reviews of Interventions, and the Preferred Reporting Items for Systematic Reviews and Meta-analysis Protocol (PRISMA),^[10]^ and the important protocol revisions will be recorded in the full review.

### 2.2 Inclusion and exclusion criteria

#### 2.2.1 Study design

Our research will be limited to randomized controlled trials (RCT). At the same time, we will weed out the repeated publications of the same study, reviews, letters, abstracts, or animal experiments.

#### 2.2.2 Participants

The study will include all patients diagnosed with COVID-19. There will be no limitation about age, region, gender, disease severity, and other factors.

#### 2.2.3 Interventions and comparators

The control group is COVID-19 patients without diabetes, while the experimental group is patients diagnosed with COVID-19 and diabetes. Both groups of patients received conventional COVID-19 treatment. The experimental group received conventional diabetes treatment recommended by the American Diabetes Association (ADA) guidelines,^[11]^ including diet, exercise, hypoglycemia, and lipid-lowering treatment, and received DPP-4 inhibitors treatment at the same time, and the control group received placebo or no treatment.

#### 2.2.4 Outcomes

The primary outcomes include mortality rate, morbidity, interleukin-6, tumor necrosis factor-alpha, clinical improvement, symptoms improvement, fasting blood glucose, 2-hour postprandial blood glucose, glycosylated hemoglobin, fasting insulin, adverse reactions, etc.

### 2.3 Study search

All reviewers decide to use a combination of title words and free words as the search strategy for this study. Electronic databases include CNKI, Wanfang, VIP, CBM database, Cochrane Library, PubMed, Web of Science, EMBASE, etc. In the meantime, for clinical trial registration and grey literature, we will manually search in Clinicaltrials.gov, the World Health Organization International Clinical Trials Registry Platform and China Conference Paper Database to make up for the lack of electronic databases. We will search each database from December 2019 to September 2020. The language of the publications will be limited to English and Chinese. We will give a detailed search process in Table 1. Adjust different search methods in the light of different Chinese and English databases.

**Table 1:**
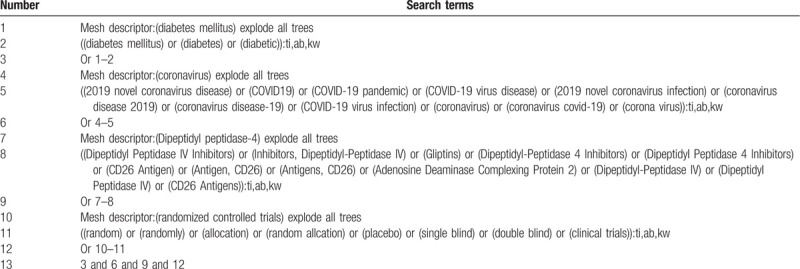
Example of Cochrane search strategy.

### 2.4 Data collection and analysis

#### 2.4.1 Selection of studies

**Figure 1:**
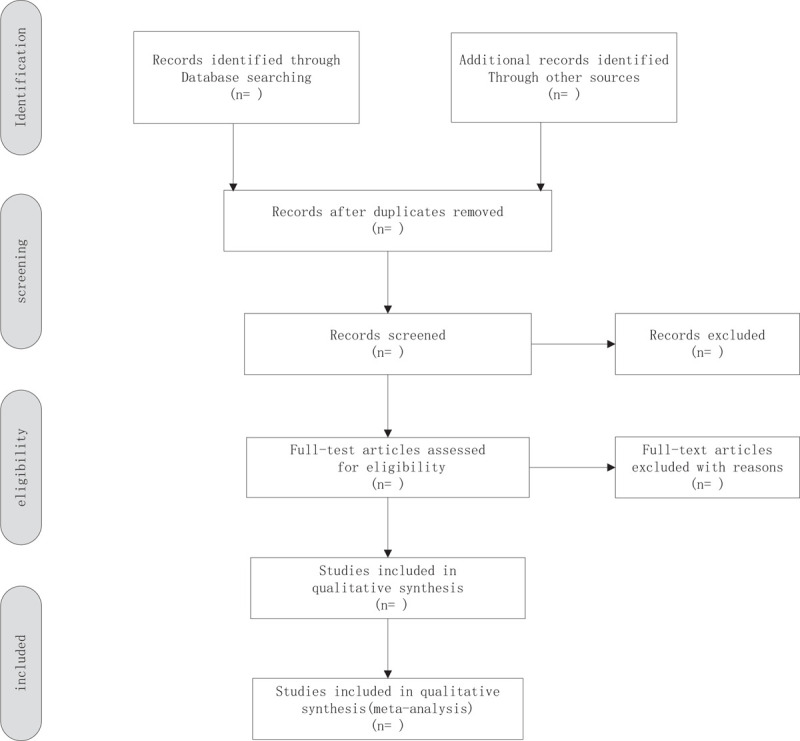
Flow chart of study selection.

We will import all the required literature into the endnote x9 software. All documents will be Preliminary screened by 2 independent reviewers by reading the title and abstract, and then the full text of the documents that meet the inclusion criteria will be carefully read to decide whether to include. In case of disagreement in the above process, this agreement will be negotiated with a third party. In addition, we will use a flowchart (Fig. 1) to show the process of exclusion causes and study selection.

#### 2.4.2 Data extraction and management

Qualified literature data will be extracted into Microsoft Excel by 2 independent reviewers. The information we want to extract is as follows: title, author, year, sample size, age, gender, course of disease, intervention measures, outcomes, and adverse reactions. If the reported data is insufficient or ambiguous, we will contact the corresponding author for complete information. If we are unable to get in touch with the author, we will exclude the study because of missing important information.

### 2.5 Risk of bias assessment

We will evaluate all the included studies according to the guidelines of Cochrane Handbook for Systematic Reviews of Interventions. Evaluation items contain the following 7 items. They are random sequence generation, allocation concealment, blinding participants and personnel, blinding evaluation of results, incomplete outcome data, selective result reporting, and other biases. The quality of each trial is classified as “low”, “high”, or “unclear”^[12]^ risk of bias. When there are disagreements, the 2 reviewers can reach a consistent conclusion through discussion or third-party consultation.

### 2.6 Data analysis

We will use Review Manager Software version 5.3 provided by Cochrane Collaboration to analyze the data. 95% RR is used to represent dichotomous data. And Continuous data will be represented by MD or SMD. When *I*^2^ < 50%, *P* > .01, it is shown that there is no statistical heterogeneity in this study, a fixed-effects model will be used; in contrast, when *I*^2^ ≥ 50%, *P* < .01, indicating that there is considered heterogeneity, a random-effects model will be used for analysis.^[13]^ In addition, according to the different causes of heterogeneity, we will further conduct subgroup or sensitivity analysis. If meta-analysis is not possible, we will conduct a descriptive analysis.

### 2.7 Subgroup analysis

We will conduct subgroup analysis based on different reasons such as age, gender, different forms of intervention, treatment process, drug dosage, etc.

### 2.8 Sensitivity analysis

In order to evaluate the robustness of the primary outcome measures, we will eliminate the low-quality studies and combine the data to assess the impact of the sample size, study quality, statistical methods, and missing data on the meta-analysis results.

### 2.9 Publication bias assessment

If there are more than 10 studies in the meta-analysis, we will evaluate the symmetry of the funnel plot to examine the publication bias and interpret the results carefully.^[14,15]^

### 2.10 Grading the quality of evidence

The entire study will evaluate the quality of evidence through the “grades of recommendations assessment, development, and evaluation (GRADE)” standard established by the WHO and international organizations. In order to be more clearer, the GRADE system divides the quality of evidence into: high, medium, low, and very low. The GRADE profiler 3.2 will be employed for analysis.

### 2.11 Patient and public involvement

Patients and the public will not be involved in this study.

### 2.12 Ethics and dissemination

Since our research is a protocol for systematic review and meta-analysis, ethical approval is not required. Our research results will also be published in peer-reviewed journals and presented at a relevant conference.

## 3 Discussion

Diabetes is a common chronic metabolic disease.^[16]^ COVID-19 is a large-scale infectious disease.^[17]^ Some studies have shown that diabetes is an independent risk factor that increases COVID-19 mortality or other adverse outcomes.^[18]^ There is currently no specific and effective drug treatment. More and more people realize that DPP-4 inhibitors may play a huge role in fighting COVID-19 combined with diabetes.^[19–20]^ However, there is no evidence-based medicine to confirm the effectiveness and safety of DPP-4 inhibitors in the treatment of COVID-19 patients with diabetes. Therefore, we are trying to conduct a meta-analysis to provide high-quality evidence for the treatment of COVID-19 diabetes patients with DPP-4 inhibitors, and to inject new impetus into the clinical response to the COVID-19 epidemic.

### 3.1 Amendments

If the research process needs to be modified, we will update our protocol.

## Author contributions

The protocol was designed by YL and HX under the guidance of HG and CX. All the authors participated in the study. The manuscript was drafted by YL and revised by HX, HG and CX. All authors approved the final manuscript before submission.

**Conceptualization:** Yan Liu, Hongyan Xie, Hong Gao and Chunguang Xie.

**Data curation:** Yan Liu, Hongyan Xie.

**Formal analysis:** Yan Liu, Hong Gao.

**Investigation:** Yan Liu, Hongyan Xie.

**Methodology:** Yan Liu, Hong Gao.

**Project administration:** Chunguang Xie.

**Software:** Yan Liu, Hong Gao.

**Visualization:** Yan Liu, Hongyan Xie.

**Writing – original draft:** Yan Liu.

**Writing – review & editing:** Chunguang Xie, Hong Gao.

## Abbreviations

Abbreviations: ADA = American Diabetes Association, COVID-19 = coronavirus disease 2019, DM = diabetes mellitus, DPP-4 = dipeptidyl peptidase-4, GRADE = Grading of Recommendations Assessment, MD = mean difference, MERS-CoV = Middle East respiratory syndrome coronavirus, PRISMA = Preferred Reporting Items for Systematic Reviews and Meta-analysis, RCT = randomized controlled trials, RR = relative risk, SARS-CoV-2 = Severe Acute Respiratory Syndrome Coronavirus 2, SMD = standard mean difference.
